# The Explosion on January 19

**Published:** 1917-01-27

**Authors:** E. W. Morris

**Affiliations:** House Governor, The London Hospital, E.


### THE EXPLOSION ON JANUARY 19.

By Mr. E. W. Morris, House Governor, The London Hospital, E.

The general facts concerning the explosion on the evening of January 19 are so well known by now that there is little left to say. Sir Henry Burdett has thought, however, that some account of that night coming from a hospital that took some share in the proceedings might be of interest to hospital workers, and therefore I very willingly send such an account.

### When the Explosion Came.

Work was proceeding quietly and as usual on that Friday evening when the explosion came. Those of us who were indoors with drawn blinds had no warning beforehand. Those, however, who happened to be crossing the grounds from one part of the hospital to another had some warning. The sky was lighted up eastwards by a red glow, which was something like that we in London have all seen when a Zeppelin is ablaze. In this case, however, the light got brighter and brighter, until at last it was like a red twilight at sunset, and the whole of the details of the hospital building could be distinctly seen. A few seconds after the moment of greatest illumination, the explosion occurred. The explosion itself was not a single bang, but more like a very short rattling clap of thunder when the storm is close overhead. That, at any rate, was what it sounded like here, although witnesses in various places describe it differently; the sound in any particular place would depend upon echoes caused by the surrounding buildings. It was very loud—much louder than any bomb, and we in the East End have learnt something about bombs.

### The Special Organisation in War-time.

We have made certain arrangements here—and probably most of the hospitals have done the same—in case of aerial attack in the immediate neighbourhood. The chief of these arrangements are as follows: The House Governor goes to the main entrance, where he may be found, and close to the telephone-room, and directs proceedings in a general way. The engineers report to him and remain ready for what may happen. Porters who are sleeping in and who have weekly fire drill also report and await orders. Orderlies, of whom we have about thirty, changeable every two months, also stand at attention. We take some trouble in teaching these orderlies certain duties they might have to carry out in case the hospital were struck by a bomb—such, for instance, as the use of hand-grenades, and also the quickest way of emptying a ward of bedridden people. These previously arranged and often rehearsed rules stood us in good stead on Friday. When the explosion occurred I ran to the main entrance and saw the chief telephone operator. She had left her instrument and was standing at the door of the room; she was certain that a bomb had dropped in the grounds close outside her window, which was broken. She went to her instrument at once and telephoned, as previously arranged, for the engineers; they appeared, however, before she got through to them, to report themselves ready for any duty. We then tried to get through to the Avenue Exchange to get some idea of what had happened, but we were unable to get any reply at all for about ten minutes.

Meanwhile a somewhat alarming smell of fire was noticed; this, however, was soon traced to the fact that soot had been driven down many of the chimneys into the rooms. Porters were drawn up awaiting orders, as were the orderlies, and the house men—house physicians and house surgeons—went to their own wards to steady their patients, also according to plan.

How the Facts reached the Hospital.

Rumours came through that Stepney Gas Works was blown up; then it was Limehouse Powder Works; then Woolwich Arsenal. After a little while we got through to the police station adjoining the Thames Police Court, and asked for some information, but they had none to give and were as much in the dark as we were. As there was nothing to be done at the moment, we all went to dinner. While dining, a call came through to me from the police, asking for medical assistance to be sent at once, if possible, giving the address. They reported a very fearful explosion at large chemical works. It was easy enough to get volunteers for such work, and in a few minutes five men and three lady doctors and some dressers were ready to start.

A Disgraceful Incident and its Pleasant Sequence.

It was not easy to get cabs, and porters were sent out to the City to find them. I should like to report that one man in Fenchurch Street absolutely refused to go, although it was pointed out to him that it was a matter of life and death to many poor men and women—he was not going for anybody. His number (not his cab's) was 2826, and anyone seeing him about London will doubtless regard him with curiosity. I did not know such fellows were to be found in England. A gentleman in a car standing near, hearing the altercation, at once drove down to the hospital and put himself at our service. We gladly accepted his offer, and he took a large car-load of dressings and instruments down to the scene of the disaster. Other taxi-drivers were much more obliging than the one I have mentioned, and two of them who had been out all day and were on their way home, but who "never refused a hospital," went down with the doctors. We sent a large supply of tourniquets, morphia syringes, and instruments for rendering first-aid, and, of course, plenty of dressings, bandages, lint, etc.

How the Injured Arrived.

After the doctors had left, Poplar Hospital rang up to say that they were full up and were passing cases on to us, and very soon they began to arrive. We keep in the stores below some hundreds of spare bedsteads ready to put up in case of emergency—one never knows what may happen in war-time. The orderlies, under the guidance of the engineers, began putting the bedsteads together, and sisters made up the beds ready for incoming patients as fast as the bedsteads were put up. We soon had many extra beds up in every ward, and others waiting in the corridors. The poor injured folk came up in every conceivable form of conveyance, but huge motor-lorries were the most important. It was bitterly cold, and we had gallons of coffee made, and this was distributed to everyone who needed it—drivers, nurses, police, porters, doctors, and orderlies.

Offer to Admit Three Hundred if Necessary.

About nine o'clock the police again rang up to ask how many we could possibly admit, and we told them any number they liked—300 if necessary. About this time St. Bartholomew's rang up, and later the Royal Free, asking if they could do anything to help us, and I gladly take the opportunity of thanking these great Charities for their courtesy and forethought. We could manage, however, but I advised them to send some doctors down to the scene of the explosion. When we had treated sixty—admitting thirty-five—there was a lull, and when our taxis returned with the doctors we heard why. The place was a furnace that it was impossible to approach. Streets of houses had become a heap of bricks, and it would be long before any more could be rescued.

A Splendid Exhibition of Unity and Service.

Many of those who came were not actually injured, but were dazed by the shock. Whitechapel Infirmary, close by, offered to take in all such as needed lodging and warmth rather than medical aid. I should like to thank the medical officer of Whitechapel Infirmary for this latest instance of his unfailing courtesy to the London. And I would thank, too, the many local general practitioners who came in that night and offered to do anything of any kind to help us. We would gladly have availed ourselves of all these offers had the hundreds we had expected actually arrived, but as so few came we were able to cope with the little extra work. We telephoned our own visiting staff that we were expecting injured, and four operating theatres were got ready to receive cases; they were soon in use. The injuries were terrible, and the exposure on that bitter night on open lorries must have been awful to those poor people, but their bearing was perfectly splendid. I do not know which I admired the more—the heroic behaviour of the injured themselves or that of the broken-hearted relatives who came with them. Five injured died during the night, and then came that dreadful time when wife came to search for husband—mother for son—among the dead and the wounded.

Three Motherless Children.

I need not go into the pathetic scenes—they are known only too well in hospital life. One at any rate I shall never forget—three little children, the eldest eight years old, carrying a little baby of a few months and leading a little toddler of four. They were not injured, only numbed with cold, and were looking for mother. They had been picked up by a kindly lorry-driver, standing dazed in a ruined street, and he had brought them along with his load of mangled bodies to the hospital.

